# Transcriptome profiling of a curdlan-producing *Agrobacterium *reveals conserved regulatory mechanisms of exopolysaccharide biosynthesis

**DOI:** 10.1186/1475-2859-11-17

**Published:** 2012-02-03

**Authors:** Anne M Ruffing, Rachel Ruizhen Chen

**Affiliations:** 1 School of Chemical and Biomolecular Engineering, Georgia Institute of Technology, 311 Ferst Drive, Atlanta, GA 30332-0100, USA

**Keywords:** Agrobacterium, Curdlan, ATCC 31749, Exopolysaccharide, Exopolysaccharide regulation, EPS transcriptional regulation

## Abstract

**Background:**

The ability to synthesize exopolysaccharides (EPS) is widespread among microorganisms, and microbial EPS play important roles in biofilm formation, pathogen persistence, and applications in the food and medical industries. Although it is well established that EPS synthesis is invariably in response to environmental cues, it remains largely unknown how various environmental signals trigger activation of the biochemical synthesis machinery.

**Results:**

We report here the transcriptome profiling of *Agrobacterium *sp. ATCC 31749, a microorganism that produces large amounts of a glucose polymer known as curdlan under nitrogen starvation. Transcriptome analysis revealed a nearly 100-fold upregulation of the curdlan synthesis operon upon transition to nitrogen starvation, thus establishing the prominent role that transcriptional regulation plays in the EPS synthesis. In addition to known mechanisms of EPS regulation such as activation by c-di-GMP, we identify novel mechanisms of regulation in ATCC 31749, including RpoN-independent NtrC regulation and intracellular pH regulation by acidocalcisomes. Furthermore, we show evidence that curdlan synthesis is also regulated by conserved cell stress responses, including polyphosphate accumulation and the stringent response. In fact, the stringent response signal, pppGpp, appears to be indispensible for transcriptional activation of curdlan biosynthesis.

**Conclusions:**

This study identifies several mechanisms regulating the synthesis of curdlan, an EPS with numerous applications. These mechanisms are potential metabolic engineering targets for improving the industrial production of curdlan from *Agrobacterium *sp. ATCC 31749. Furthermore, many of the genes identified in this study are highly conserved across microbial genomes, and we propose that the molecular elements identified in this study may serve as universal regulators of microbial EPS synthesis.

## Background

Microbial exopolysaccharides (EPS) are a class of natural products with stunning chemical diversities, which are comprised of a combination of constituent sugars, many different types of glycosidic linkages, stereochemistry, and additional chemical modifications on the sugars. In parallel to their chemical diversity, they display diverse physical properties that make them useful in many different applications. For their microbial producers, EPS serve important physiological functions, including cell adhesion, biofilm formation, symbiosis, protection from desiccation, and evasion of the human immune system [1]. Despite the chemical, physical, and physiological diversity they display, an outstanding common feature is that they are synthesized under adverse growth conditions. Often, their synthesis is triggered by a nutritional stress, and significant accumulation is only observed in the stationary phase [2]. As such, it is conceivable that there may be shared mechanisms by which microbes translate environmental cues into intracellular signals and subsequently activate the machinery for EPS synthesis. Another intriguing fact is that EPS synthesis entails a significant input of cellular resources at a time when these resources are limited. There may be unifying mechanisms by which cells channel limited resources for EPS synthesis. As a first step to elucidate these possible conserved mechanisms, we apply transcriptome analysis to identify molecular components that may play roles in signal transduction of nutritional limitation, energy storage, and stress responses. We use curdlan synthesis in *Agrobacterium *sp. ATCC 31749 as our model system.

*Agrobacterium *sp. ATCC 31749 is a natural producer of curdlan EPS and is employed for large-scale curdlan production. Curdlan is used in a wide-range of applications including the production of superworkable concrete, as a gelling agent in the food industry, and as a main component of potential anti-tumor and anti-HIV treatments [3]. Despite the economic importance of curdlan, relatively little is known regarding the genetics and regulation of curdlan synthesis in ATCC 31749. Four genes are required for curdlan biosynthesis: *crdA, crdS, crdC*, and *crdR *[4]. Curdlan synthesis is in response to nitrogen limitation, and two components of the nitrogen signaling cascade, NtrB and NtrC, were reported to be essential for curdlan biosynthesis [3]. In other well-characterized microorganisms like *E. coli*, nitrogen-limited processes are transcriptionally regulated via the nitrogen signaling cascade, in which NtrC activates transcription via the σ^N^-containing form of RNA polymerase [5]. If this model holds true for curdlan synthesis, the sigma factor σ^N ^would play an important role in the transcriptional regulation of the curdlan operon. Our recent genome sequencing of ATCC 31749 [6] suggests the presence of a bacterial organelle, known as an acidocalcisome, in this strain, similar to the closely related *Agrobacterium tumefaciens *[7]. This is potentially relevant to curdlan synthesis as the organelle is specifically involved in the storage and metabolism of cellular polyphosphate (polyP) [8]. Kornberg and his coworkers suggested that polyP, linear polymers of tens or hundreds of orthophosphate residues linked by high-energy phosphoanhyride bonds, can substitute for ATP, among other functions, in a variety of cellular processes [9]. They later found that polyP levels are co-regulated with AlgR, a regulatory protein for the synthesis of alginate, an EPS synthesized by *Pseudomonas aeruginosa *[10]. Moreover, they found a link between polyP accumulation and nitrogen limitation [11]. The connection of EPS synthesis to polyP, however, is complicated by the fact that polyP may serve as not only a potential ATP substitute, but also as a regulator of cellular functions involved in stress response and cell survival, which may also be important for EPS synthesis [12]. The genome sequence also indicates the presence of numerous GGDEF-containing proteins associated with the synthesis and degradation of bis-(3',5')-cyclic-dimeric-guanosine monophosphate (c-di-GMP), previously shown to play a role in regulating EPS synthesis [13], suggesting another potential conserved mechanism of regulation for EPS biosynthesis.

The understanding of EPS biosynthesis regulation is most advanced for alginate synthesis in *Pseudomonas aeruginosa*, owing to the importance of alginate in pathogenesis. The current model, as reviewed by Rehm and Valla, depicts a complex network that involves multiple environmental sensing and signal transduction elements, regulatory proteins both global (catabolite activator protein) and specific to alginate (AlgR), and a specific sigma factor (AlgU) [14]. We expect the regulatory circuits of curdlan biosynthesis will also be multifaceted, and thus, the application of systems biology tools will be most fruitful in identifying the factors involved in the process. In this work, we first use transcriptome profiling to identify genes that are differentially expressed during curdlan synthesis and subsequently carry out targeted gene knockouts to investigate their roles in the transcriptional regulation of curdlan production. We focus on the signal transduction of nitrogen limitation, the potential role of acidocalcisomes for energy provision, and the stringent response, as we believe these aspects, to some degree, are shared by other microbial EPS.

## Results

### Transcriptome analysis of ATCC 31749

**Figure 1:**
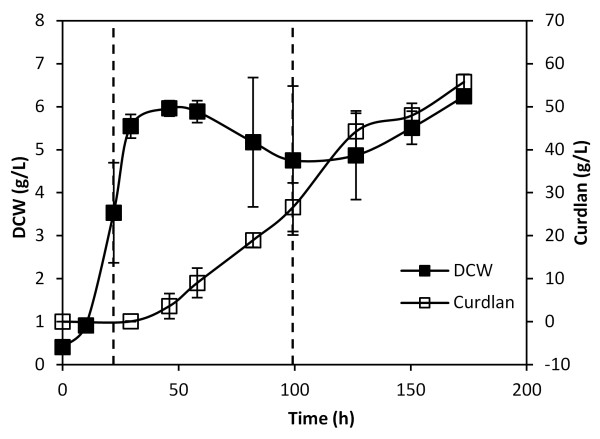
**Dry cell weight (DCW) and curdlan concentration during cultivation of ATCC 31749**. Vertical lines indicate sampling times for gene expression analysis. Error bars represent the standard deviation of 3 biological replicates.

Like many other EPS, curdlan biosynthesis is triggered by nutrient limitation, specifically nitrogen starvation [15]. To investigate the regulation of curdlan synthesis, a custom DNA microarray was designed using the draft genome sequence of ATCC 31749, and transcriptome analysis was conducted on samples taken at 22 hours, during the exponential growth phase without curdlan production, and at approximately 70 hours after nitrogen depletion, during curdlan production (Figure 1). The microarray data was deposited in Gene Expression Omnibus (GEO accession number: GSE32576). A total of 2,456 genes had more than a 2-fold change in gene expression level during curdlan production (Table 1), of which 985 were up-regulated and 1,471 were down-regulated. Notably, the curdlan biosynthesis operon (*crdASC*) was up-regulated by up to 100-fold (Table 2), providing the first experimental evidence of curdlan transcriptional regulation. Genes with significant expression changes were grouped according to function (Table 1). A majority of the up-regulated genes (55%) code for hypothetical proteins of unknown function, which contributes to the difficulty in elucidating the regulatory mechanism of curdlan biosynthesis. Besides hypothetical proteins, genes associated with transporters, metabolism and cofactor synthesis, and regulation also experienced significant changes (Table 1), suggesting extensive responses to the condition that triggers curdlan production.

**Table 1:** Functional distribution of genes up and down-regulated during curdlan biosynthesis

Functional Category	Up-regulated		Down-regulated	
	
	# of genes	%	# of genes	%
Cell replication and division	43	4.4	82	5.6

Membrane and lipid metabolism	18	1.8	69	4.7

Protein synthesis and degradation	31	3.1	104	7.1

RNA-associated proteins	4	0.4	74	5.0

Transporters	86	8.7	210	14.3

Metabolism and cofactor synthesis	111	11.3	381	25.9

Regulation	105	10.7	101	6.9

Stress response/cell protection	27	2.7	26	1.8

EPS synthesis and degradation	8	0.8	24	1.6

Hypothetical proteins	546	55.4	395	26.9

Phage/invasion/virulence proteins	6	0.6	5	0.3

**Total**	**985**	**100.0**	**1471**	**100.0**

**Table 2:** Gene expression changes during curdlan production for select genes with potential influence on curdlan biosynthesis

Gene ID	Fold Change	P-value	Gene Symbol	Description
*Curdlan biosynthesis*				

AGRO_1847	99.0	7.8E-4	*crdA*	hypothetical protein Atu3057

AGRO_1848	94.8	1.1E-3	*crdS*	β1,3-glucan synthase catalytic subunit

AGRO_1849	65.8	9.1E-5	*crdC*	hypothetical protein Atu3055

AGRO_1729	47.5	4.8E-5	-	ECF family RNA polymerase sigma factor

*Nitrogen signaling cascade*				

AGRO_1033	5.2	1.4E-2	*glnK*	nitrogen regulatory protein PII

AGRO_4555	2.0	1.8E-2	*nifR*	nitrogen regulation protein

AGRO_0407	1.9	8.3E-3	*rpoN*	RNA polymerase factor sigma-54

AGRO_4554	-1.0	8.1E-1	*ntrB*	two component sensor kinase

AGRO_4553	-2.0	9.4E-2	*ntrC*	two component response regulator

AGRO_0420	-5.8	1.0E-3	*glnD*	PII uridylyl-transferase

*c-di-GMP biosynthesis*				

AGRO_3967	5.7	3.2E-3	-	GGDEF domain protein

AGRO_0636	2.3	5.7E-2	-	GGDEF domain protein

AGRO_0033	2.2	4.2E-2	-	GGDEF domain protein

*Acidocalcisome-associated*				

AGRO_5348	2.4	5.1E-4	*chaA*	Ca^2+^/H^+ ^antiporter

AGRO_2774	1.9	3.3E-3	*ppx1*	exopolyphosphatase

AGRO_2974	1.8	3.0E-1	*aqpZ*	aquaporin Z

AGRO_2553	1.1	4.4E-1	*ppk*	polyphosphate kinase

AGRO_1846	1.1	6.7E-1	-	Na^+^/H^+^antiporter

AGRO_0927	-2.2	6.8E-4	*ppa*	Inorganic pyrophosphatase

GRO_2552	-3.6	2.5E-3	*ppx2*	exopolyphosphatase

AGRO_2518	-6.9	4.5E-3	*rrpP*	membrane-bound proton-translocating pyrophosphatase

*Stringent response*				

AGRO_1497	3.9	1.6E-3	*relA/spoT*	GTP pyrophosphohydrolase/synthetase

**Figure 2:**
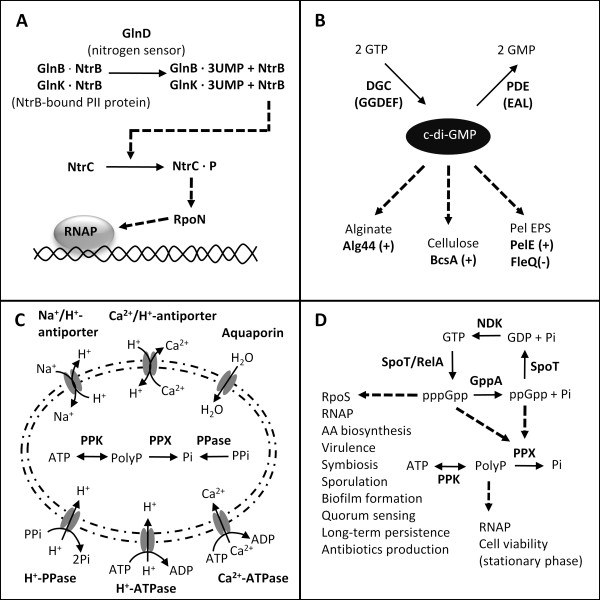
**Schematic of possible regulatory mechanisms and metabolic factors affecting curdlan biosynthesis: nitrogen signaling cascade (A), nucleotide-based second messenger c-di-GMP (B), acidocalcisomes (C), and stringent response (D)**.

Many mechanisms may regulate or influence curdlan biosynthesis, including the nitrogen signaling cascade, nucleotide-based second messengers such as c-di-GMP, energy storage in acidocalcisomes, and the stringent response (Figure 2). Differentially expressed genes associated with these potential regulatory mechanisms were selected for further experimental analysis (Table 2).

### Transcriptional regulation of curdlan production under nitrogen-limitation

**Figure 3:**
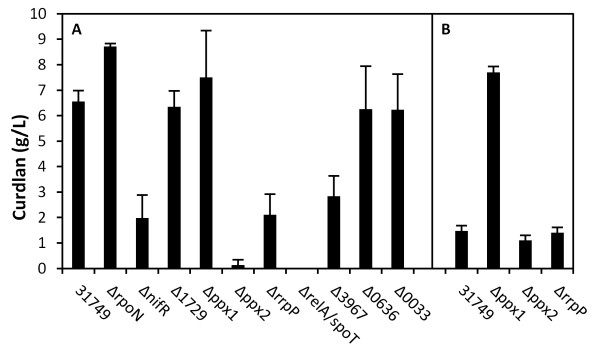
**Curdlan biosynthesis in ATCC 31749 and gene knockout mutants after 24 hours of cultivation in nitrogen-free media using stationary phase (A) and late exponential phase (B) cells**. Error bars represent the standard deviation of 3 biological replicates.

In bacteria and euryarchaeota, most nitrogen regulatory mechanisms are centrally controlled by a conserved and ancient set of nitrogen sensor proteins called PII [5] (Figure 2), and the signal is transmitted by a two component signal transduction mechanism involving NtrB and NtrC. In the cascade, NtrC is phosphorylated by NtrB under nitrogen-deplete conditions. In turn, phosphorylated NtrC binds to the RpoN sigma factor to initiate transcription [5]. Previously, NtrB and NtrC, were shown to be necessary for curdlan biosynthesis in ATCC 31749 [3]. The genome of this strain encodes a full complement of genes for nitrogen sensing and signal transduction, but curiously, the *ntrBC *operon also contains *nifR*, whose function is unknown but appears as the first gene of the *ntrBC *operon in nitrogen-fixing α-proteobacteria. Thus, NifR appears to be associated with nitrogen fixation, yet, the draft genome of ATCC 31749 lacks a nitrogenase, rendering this strain incapable of nitrogen fixation. The transcriptome analysis showed that expression of *nifR *increased 2-fold during curdlan synthesis (Table 2). To determine whether *nifR *encodes a protein that is relevant for curdlan biosynthesis, a knockout mutant was generated. As shown in Figure 3A, the *nifR *mutant was able to synthesize curdlan, yet the production of curdlan was reduced to 30% of the wild type production. While we cannot rule out polar effects from deletion of *nifR *on *ntrBC *transcription, these results confirm the link between curdlan biosynthesis and nitrogen-limited regulation.

To determine if the classical mechanism of the nitrogen signaling cascade regulates transcription of the curdlan synthesis operon, the sigma factor *rpoN *was targeted for gene knockout. Surprisingly, the *rpoN *mutant not only synthesized curdlan (Figure 3A), but it produced over 30% more curdlan than the wild type. This result indicates that the regulation of curdlan biosynthesis is RpoN-independent, implicating an alternative sigma factor for transcriptional activation of the curdlan synthesis operon. Furthermore, a 30% increase in curdlan production for Δ*rpoN *suggests that sigma factor competition may occur, whereby deletion of RpoN reduces sigma factor competition for RNA polymerase and allows for increased curdlan biosynthesis through the yet unknown alternative sigma factor [16]. One plausible candidate sigma factor is AGRO_1729, an extracytoplasmic function (ECF) sigma factor, which displayed a 47-fold increase in gene expression during curdlan production (Table 2). As shown in Figure 3A, the AGRO_1729 knockout mutant produced curdlan at levels similar to the wild-type, suggesting its minimal involvement in regulating curdlan synthesis. While the sigma factor responsible for the transcriptional regulation remains elusive, the data presented here confirmed the role of *ntrBC *operon in the signal transduction but the mechanism involving the *ntrBC *operon seems to be unique for curdlan synthesis in ATCC 31749.

### Curdlan synthesis is influenced by the nucleotide second messenger, c-di-GMP

Exopolysaccharides, such as cellulose, alginate, and Pel polysaccharide, are regulated by the nucleotide second messenger, c-di-GMP. C-di-GMP acts as an allosteric regulator of cellulose and alginate biosynthesis, and it serves as both an allosteric and transcriptional regulator of Pel EPS in *Pseudomonas aeruginosa *[13]. C-di-GMP is synthesized by diguanylate cyclases which contain a conserved GGDEF motif as the catalytic active site. The genome sequence of ATCC 31749 contains 31 predicted genes coding for GGDEF domain proteins which may control c-di-GMP levels and potentially regulate curdlan biosynthesis. Only 3 GGDEF domain proteins had more than a 2-fold up-regulation in gene expression under nitrogen limitation (Table 2); the corresponding genes were targeted for deletion.

The AGRO_3967 mutant showed a 57% decrease in curdlan production (Figure 3A), suggesting that c-di-GMP may regulate curdlan biosynthesis. The other GGDEF domain mutants (Δ0033 and Δ0636), however, produced curdlan at levels similar to that of the wild-type. These results correlate with the measured gene expression levels. AGRO_3957 displayed the largest increase in gene expression (5.7-fold), and its associated mutant had impaired curdlan production. On the other hand, AGRO_0033 and AGRO_0636 not only had less of an increase in gene expression (< 2.4-fold), but the statistical significance of increased gene expression was low for these GGDEF domain genes (p-values > 0.042). Accordingly, curdlan production was unaffected in the corresponding AGRO_0033 and AGRO_0636 knockout mutants. Overall, these results suggest c-di-GMP levels in ATCC 31749 affect curdlan biosynthesis, and more specifically, c-di-GMP synthesis via AGRO_3967 is important for high curdlan production.

### Acidocalcisomes and PolyP metabolism influence curdlan synthesis

Curdlan biosynthesis is an energy-intensive process, requiring two high-energy molecules for each nascent glycosidic bond. In fact, energy availability was shown to be a limiting factor of curdlan biosynthesis in ATCC 31749 [17]. One potential source of energy is polyP, a phosphate polymer containing many high-energy bonds. In *Agrobacterium tumefaciens*, a close relative of ATCC 31749, polyP accumulates in intracellular organelles known as acidocalcisomes [7]. The ATCC 31749 genome contains many genes associated with acidocalcisomes, including those listed in Table 2. The influence of acidocalcisomes and polyP on curdlan biosynthesis was investigated.

The accumulation of polyP in acidocalcisomes is enhanced under stationary phase or stress conditions [18]. In *E. coli*, for example, the level of polyP is low in the exponential phase and increases up to 1000-fold upon amino acid starvation [19]. Nitrogen limitation also elicits a high level of polyP accumulation [11]. Both nitrogen depletion and the stationary phase are also required for optimal curdlan production, providing the first clue that polyP may be related to curdlan production. Cells harvested from stationary phase produced 6.5 g/L of curdlan after 24 hours of cultivation under nitrogen deplete conditions, whereas cells harvested from exponential phase only produced 1.5 g/L under the same conditions (Figure 3). The level of poly P is influenced by the activity of exopolyphosphatase (PPX), an enzyme that hydrolyzes a phosphate molecule from the polymer chain of polyP. As shown in Figure 3B, knockout of *ppx1 *(encoding a putative exopolyphosphatase) led to significant increase in curdlan synthesis compared to wild type cultures for cells harvested from the exponential phase. Eliminating exopolyphosphatase activity allows polyP to accumulate during the exponential phase, subsequently leading to curdlan production at the high levels traditionally observed only with stationary phase cultures. In contrast, knockout of *ppx1 *has no effect on curdlan synthesis for stationary phase cells (Figure 3A), consistent with the expectation that exopolyphosphatase activity is already low in the stationary phase due to the high level of (p)ppGpp, an inhibitor to PPX which typically accumulates in the stationary phase [19]. These results suggest that polyP may serve as an important energy source for curdlan biosynthesis in ATCC 31749. It is important to note that the genome encodes two PPXs but experimental evidence showed PPX2 behaves quite differently from PPX1 and does not likely use polyP as substrate.

Previous studies showed strong correlation of pH and curdlan synthesis, with pH 5.5 being optimal for curdlan synthesis [20], significantly lower than its optimum for growth (pH 7.0). In addition to the phosphate and energy storage functions associated with polyP accumulation, acidocalcisomes could also influence curdlan synthesis through pH regulation, as the organelle is also known to play important role in maintenance of intracellular pH [8]. The putative acidocalcisome gene with a role in regulating pH is *rrpP*, a membrane-bound proton-translocating pyrophosphatase, which showed nearly a 7-fold drop in gene expression under curdlan-producing conditions (Table 2). Additionally, gene knockout of *rrpP *led to nearly a 70% decrease in curdlan production compared to the wild type (Figure 3A), indicating that proton translocation between the cytosol and acidocalcisome, mediated by RrpP, is important for optimum curdlan synthesis.

Taken together, the experimental data from this study provides the first evidence to link the acidocalcisome with curdlan synthesis through both high energy provision and pH regulation.

### The stringent response regulates transcription of the curdlan synthesis operon

In addition to its potential as a high energy storage compound, polyP accumulation is an essential element of the stringent response (Figure 2). The stringent response was first studied in *E. coli *as a response to amino acid starvation, but it has since been linked with other stress conditions including nitrogen limitation [11,21]. During the stringent response, guanosine pentaphosphate (pppGpp) and guanosine tetraphosphate (ppGpp), collectively known as (p)ppGpp, accumulate within the cell. Besides acting as allosteric inhibitors of exopolyphosphatase, (p)ppGpp can directly influence gene transcription and translation [21].

**Figure 4:**
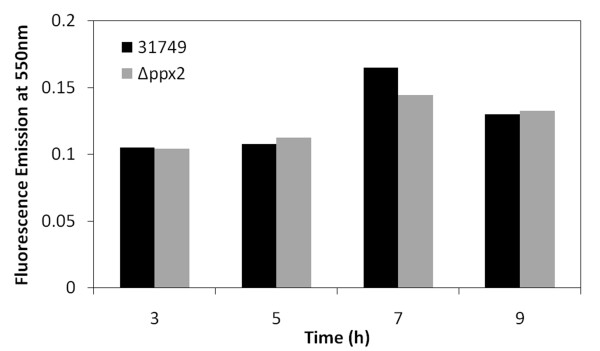
**Relative polyP levels in wild type (31749) and Δ*ppx2 *during exponential growth**.

**Figure 5:**
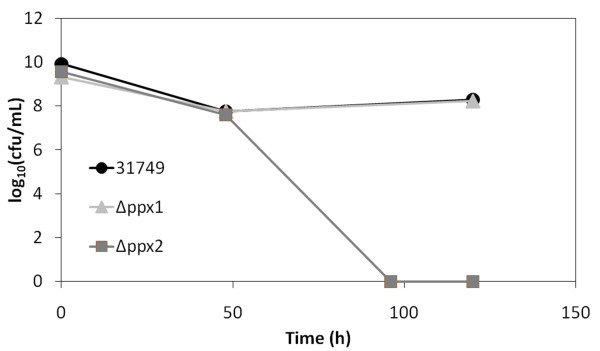
**Cell viability of wild type (31749), Δ*ppx1*, and Δ*ppx2 *during curdlan production measured by colony forming units (cfu)**.

The previous section on polyP metabolism describes two predicted *ppx *genes in ATCC 31749, yet experimental evidence suggests only one gene product (*ppx1*) has exopolyphosphatase activity. The other predicted exopolyphosphatase gene (*ppx2*) likely has phosphatase activity, yet it may utilize pppGpp as substrate rather than polyP. From its amino acid sequence, *ppx2 *has two predicted catalytic domains, one for exopolyphosphatase (PPX) activity and one for guanosine pentaphosphate phosphohydrolase (GppA) activity. As additional evidence that PPX2 does not exhibit exopolyphosphatase activity, the level of polyP during growth of Δ*ppx2 *was not enhanced compared to polyP levels in the wild type (Figure 4). If PPX2 functions primarily as a GppA, *ppx2 *deletion would yield high levels of pppGpp and low levels of ppGpp. Stationary phase cultures of Δ*ppx2 *show nearly a 98% reduction in curdlan biosynthesis (Figure 3A), suggesting that ppGpp may be the active form of (p)ppGpp for the stringent response in ATCC 31749. Since (p)ppGpp only accumulates under stationary phase conditions, knockout of *ppx2 *is not expected to influence curdlan production in exponential phase cultures. In agreement with this, exponential phase cultures of Δ*ppx2 *produced curdlan at levels similar to the wild type (Figure 3B). Interestingly, the *ppx2 *mutant also showed reduced cell viability under nitrogen limitation (Figure 5). This behavior is similar to that observed in both polyP- and ppGpp-deficient *E. coli *cells which are not able to survive under stationary phase conditions [22,23]. The reduced viability of Δ*ppx2 *during stationary phase suggests that the ppGpp form of (p)ppGpp is important for activating stress responses in ATCC 31749.

The stringent response factor (p)ppGpp is synthesized in *E. coli *by two different enzymes: RelA, a (p)ppGpp synthetase, and SpoT, a bifunctional (p)ppGpp synthetase/ppGpp 3'-phosphohydrolase [16]. Using *E. coli *RelA and SpoT sequences (Accession numbers: NP_417264 and ACB04699) as query, BLASTP identified AGRO_1497 as the only RelA and SpoT homolog in ATCC 31749, having 31% and 37% identity with the *E. coli *sequences respectively. It appears that this single *relA/spoT *homolog controls both (p)ppGpp synthesis and degradation. Deletion of *relA/spoT *completely eliminates curdlan synthesis in ATCC 31749 under nitrogen-limited conditions (Figure 3A). A reduction in curdlan biosynthesis may be explained by a decreased level of polyP due to elimination of the PPX inhibitor, (p)ppGpp. Yet, curdlan production is not simply reduced in the *relA/spoT *mutant; it is completely eliminated, suggesting the effect of (p)ppGpp extends beyond its inhibition of PPX. Based on the results from both the *ppx2 *(putative GppA) and *relA/spoT *mutants, the stringent response signal, (p)ppGpp, is clearly essential for curdlan biosynthesis.

(p)ppGpp is a global regulator of gene expression in *E. coli *and other bacteria [16]. Thus, to determine if (p)ppGpp is involved in the transcriptional regulation of the curdlan synthesis operon (*crdASC*), gene expression levels of curdlan synthase (*crdS*) in the *relA/spoT *and *ppx2 *mutant strains were compared to wild type. Under nitrogen limitation, Δ*relA/spoT *cultures exhibited *crdS *expression levels that were up to 57-fold lower than in wild type cultures. This demonstrates that (p)ppGpp is involved in the activation of transcription of curdlan synthase genes. As discussed above, knockout of the putative GppA, *ppx2*, led to impaired curdlan production; however, *crdS *expression levels were only reduced up to 2-fold in Δ*ppx2 *compared to wild type. This may indicate that the two forms of (p)ppGpp, pppGpp and ppGpp, have differential mechanisms of regulating curdlan synthesis.

## Discussion and conclusions

Microbial EPS are often synthesized in response to a nutritional stress, with nitrogen limitation being a common environmental cue. As EPS synthesis entails a significant investment in terms of cellular energy and other resources under a condition when resources and energy are scarce, extensive re-organization of metabolic activity is expected and sophisticated regulatory mechanisms are needed to respond to environmental triggers. The common trigger of nutrient limitation for their synthesis and their association with adverse growth conditions suggest some unifying scheme of EPS regulation. Yet, our understanding is very limited about how their biosynthesis is regulated at the molecular level. In this study, we probe the regulation mechanisms of curdlan synthesis with a focus on those potentially conserved mechanisms. The transcriptome profiling along with genetic, biochemical, and physiological analysis reveals a multifaceted network of regulation for curdlan biosynthesis, including RpoN-independent nitrogen regulation, c-di-GMP, acidocalcisomes, polyP metabolism, and the stringent response.

First, the transcriptome analysis showed that upon nitrogen depletion, the curdlan synthesis operon was up-regulated by up to 100-fold, thus transcriptional regulation plays a prominent role in its biosynthesis. As curdlan synthesis is activated in response to nitrogen depletion, the involvement of the nitrogen signaling cascade is expected. The signal transduction components, NtrB and NtrC, were previously identified as essential components for curdlan synthesis [3], and this was confirmed in this study. Additionally, this study uncovered some unexpected details of how nitrogen signaling affects curdlan synthesis. The operon structure for *ntrBC*, having *nifR *as the first gene, is unusual for a non-nitrogen fixer, and *nifR *deletion was found to reduce curdlan production. We also show that the RpoN sigma factor is not involved, implicating an alternative sigma factor in the regulation. This is a significant departure from the archetype nitrogen regulation, in which RpoN is required for transcription of the regulated genes and NtrC serves as the activator. RpoN-independent transcriptional regulation has been shown to operate extensively in only one other microorganism, *Rhodobacter capsulatus*, a photosynthetic bacterium and a nitrogen fixer [24]. Interestingly, *R. capsulatus *also contains a *nifR*-like gene (*nifR3*) in the same operon as *ntrBC *[25], suggesting a connection between the *ntrBC *operon structure and its RpoN independence. Future studies to identify the elusive sigma factor responsible for the regulation holds the key to understand this new mode of nitrogen signaling.

The genome sequence of ATCC 31749 suggests the presence of aciocalcisomes. This study provides the first evidence that metabolism within the organelle influences curdlan biosynthesis through energy storage in the form of polyP and maintenance of intracellular pH. The reduced curdlan produced by Δ*rrpP *suggests that the membrane-bound proton-translocating pyrophosphatase (RrpP) plays an essential role in acidocalcisome-mediated intracellular pH regulation. By eliminating polyP degradation via *ppx1 *deletion, high curdlan synthesis could be triggered in exponential phase cells, clearly establishing the role of polyP in curdlan biosynthesis. The contribution of polyP as an energy source in curdlan biosynthesis, however, is complicated by the regulatory role of polyP in the stringent response.

Despite the fact that many EPS are produced under nutritionally stressed conditions which likely trigger a stringent response, bacterial EPS synthesis have rarely been studied in the context of the stringent response. In this study, we show that curdlan synthesis is abolished in cells lacking the gene responsible for synthesis of the stringent response signal (p)ppGpp. This indicates that curdlan synthesis requires the stringent response. Further analysis by qRT-PCR showed that the curdan synthesis operon was down-regulated by 57-fold in the *relA/spoT *deletion mutant. Thus without the stringent response, up-regulation of the curdlan operon could not be elicited. This is a significant finding as it convincingly establishes requirement of the stringent response for the transcriptional up-regulation of an EPS operon. Only one previous report described a relationship between the stringent response and EPS synthesis [26]. However, in this report, the *Sinorhizobium meliloti *mutant incapable of synthesizing the effector molecule of stringent response by knocking out the only *relA/spoT *gene (the same approach as used in this study), produced more succinoglycan, an EPS, and the succinoglycan operon was significantly up-regulated in the mutant. Thus, in this case, ppGpp negatively regulates succinoglycan synthesis, which is significantly different from the complete dependence of curdlan synthesis on the presence of the effector molecule, (p)ppGpp. Hence, our data suggest a novel type of EPS regulation as a direct effect of the stringent response, which has not been reported. The molecular details of activation, however, await further elucidation. As reviewed by Srivatsan and Wang, two models of activation were proposed for (p)ppGpp [21]. In direct activation, the effector molecule, (p)ppGpp, increases transcription from cognate promoters by acting on RNA polymerase, whereas indirect activation involves an alternative sigma factor. Much of the understanding of the stringent response, however, is based on the studies with *E. coli*, in which the stationary phase sigma factor, RpoS, plays a central role in stringent response [23]. Unfortunately, the *E. coli *model is unlikely to be applicable here as the ATCC 31749 genome does not encode a corresponding stationary phase sigma factor. Additionally, the ATCC 31749 genome contains only one *relA/spoT *homolog and two predicted exopolyphosphatases, signifying a significant departure from the *E. coli *system.

Additional data were obtained, which demonstrated the influence of the nucleotide second messenger, c-di-GMP, on curdlan synthesis. C-di-GMP is synthesized by diguanylate cyclases which contain a conserved GGDEF motif as the catalytic active site [13]. The genome sequence of ATCC 31749 contains 31 predicted genes coding for GGDEF domain proteins, of which three GGDEF domain proteins had more than a 2-fold up-regulation in gene expression under nitrogen limitation (Table 2). Deletion of AGRO_3967, encoding an up-regulated GGDEF domain protein, yielded a 57% decrease in curdlan production (Figure 3A), suggesting that c-di-GMP may regulate curdlan biosynthesis, thus adding another factor to the already complicated regulatory network.

The use of systems biology tools in this study identified many components important to the transcriptional regulation of curdlan synthesis, including the *ntrBC *operon in nitrogen signal transduction, polyP, (p)ppGpp, and c-di-GMP. Subsequent experiments with gene knockout mutants and qRT-PCR revealed some aspects that are unique to curdlan synthesis and the producer microorganism. These include RpoN-independent activation of the curdlan operon and the connection of curdlan synthesis to the metabolic activity of a bacterial organelle. We also present convincing evidence that curdlan biosynthesis is involved in the stringent response. While these three aspects appear to be unique to curdlan, the components identified, NtrBC, polyP, (p)ppGpp, and c-di-GMP, are present in most microorganisms that synthesize EPS. Thus, it is reasonable to expect some common features to emerge. Indeed, Kornberg suggested that polyP may have served as an ATP-alternative energy source [9], and subsequent work from his lab confirmed the role of polyP in alginate synthesis in *P. aeruginosa *[10], and in a separate work, demonstrated a connection between polyP accumulation, nitrogen limitation, and the stringent response [11]. Additionally, the stringent response in *P. aeruginosa *was recently shown to regulate quorum sensing [27], which affects synthesis of the EPS alginate [28], providing another link between the stringent response and EPS biosynthesis. Thus, polyP, a ubiquitous molecule accumulated in response to nitrogen limitation and in the stationary phase, plays a central role in the regulation of EPS synthesis and may additionally serve as an energy source. Our findings from this study further support this notion. In reference to the alginate model as reviewed by Rehm and Valla, a sigma factor (AlgU) and regulatory protein (AlgR), are central to integrate the molecular components influencing alginate biosynthesis [14]. Data presented here indicate that an unidentified sigma factor is involved in the regulation, and previous studies implicate the presence of a regulatory protein (CrdR) required for curdlan synthesis [3]. Further studies with CrdR and identification of the yet unknown sigma factor involved in the transcriptional regulation will be important to integrate the information uncovered in this work. While we believe each regulatory network may differ in some specific details, NtrC, polyP metabolism, and stringent response are aspects that may provide a basis for unified mechanisms of EPS regulation.

## Methods

### Bacterial strains and plasmids

The bacterial strains and plasmids used in this study are listed in Table 3. For gene knockout, an internal fragment of the target gene (200-500 bp) was cloned and inserted into the *SacI *and *HindIII *sites of the suicide vector, pUCP30T, following the method outlined in [29]. The primers used for cloning the internal fragments are listed in Table 4. The knockout plasmids were transformed via electroporation using the BioRad micropulser Agr program. Knockout mutants were selected by screening on LB-agar plates containing 50 μg/mL of gentamicin. Successful gene KO was confirmed with PCR using primers that span the junction between the chromosome and inserted KO plasmid (Table 4).

**Table 3:** Bacterial strains and plasmids used in this study

Strain/Plasmid	Description	Source
JM109	*E. coli *K12 strain used for cloning	Promega

ATCC 31749	Curdlan-producing *Agrobacterium *sp. (wild-type)	ATCC

ATCC 31749Δ*nifR*	ATCC 31749 mutant with gene knockout of *nifR*	This study

ATCC 31749Δ*ppx1*	ATCC 31749 mutant with gene knockout of *ppx1 *(AGRO_2774)	This study

ATCC 31749Δ*ppx2*	ATCC 31749 mutant with gene knockout of *ppx2 *(AGRO_2552)	This study

ATCC 31749Δ*rpoN*	ATCC 31749 mutant with gene knockout of *rpoN*	This study

ATCC 31749Δ*rrpP*	ATCC 31749 mutant with gene knockout of *rrpP*	This study

ATCC 31749ΔrelA/*spoT*	ATCC 31749 mutant with gene knockout of AGRO_1497	This study

ATCC 31749Δ*0033*	ATCC 31749 mutant with gene knockout of AGRO_0033	This study

ATCC 31749Δ*0636*	ATCC 31749 mutant with gene knockout of AGRO_0636	This study

ATCC 31749Δ*1729*	ATCC 31749 mutant with gene knockout of AGRO_1729	This study

ATCC 31749Δ*3967*	ATCC 31749 mutant with gene knockout of AGRO_3967	This study

DH5α/pUCP30T	*E. coli *strain containing suicide vector	H. Schweitzer

pGEM-T easy	Vector used for cloning internal gene fragments	Promega

pUCP30T-*nifR*	Gene knockout plasmid for interruption of *nifR*	This study

pUCP30T-*ppx1*	Gene knockout plasmid for interruption of *ppx1 *(AGRO_2774)	This study

pUCP30T-*ppx2*	Gene knockout plasmid for interruption of *ppx2 *(AGRO_2552)	This study

pUCP30T-*rpoN*	Gene knockout plasmid for interruption of *rpoN*	This study

pUCP30T-*rrpP*	Gene knockout plasmid for interruption of *rrpP*	This study

pUCP30T-*spoT*	Gene knockout plasmid for interruption of *spoT*	This study

pUCP30T-*0033*	Gene knockout plasmid for interruption of AGRO_0033	This study

pUCP30T-*0636*	Gene knockout plasmid for interruption of AGRO_0636	This study

pUCP30T-*1729*	Gene knockout plasmid for interruption of AGRO_1729	This study

pUCP30T-*3967*	Gene knockout plasmid for interruption of AGRO_3967	This study

**Table 4:** Primers used in this study.

Target Gene	Forward/Reverse	Primer Sequence
*Internal gene fragment*		
*nifR*	F	5'- GCAAGCTTCCCAATCCGCCCGTCCCTC - 3'
	
	R	5'- CAGAGCTCGAAATGGTCGCCAGCCGC - 3'

*ppx1 *(AGRO_2774)	F	5'- CTAAGCTTGCAGCGAGGTCCAGTGGGTG - 3'
	
	R	5'- CAGAGCTCCAGTTCCGGGTCGTCGATG - 3'

*ppx2 *(AGRO_2552)	F	5'- CTAAGCTTGCTCCAATCCGCCAGTTCGC - 3'
	
	R	5'- CAGAGCTCGGTCGGTGGTACATGGCGAA - 3'

*rpoN*	F	5'- GCAAGCTTCATCGAATCGTAACCCGCG - 3'
	
	R	5'- GCGAGCTCAGCGACCACCTCAATCAG - 3'

*rrpP*	F	5'- GTAAGCTTGATCAGCGCCGTCACGACAAG - 3'
	
	R	5'- CAGAGCTCCCGCGCAACCCTGCTACCAT - 3'

*relA/spoT *(AGRO_1497)	F	5'- GTAAGCTTGTATGCTCCAGGAACTCTTC - 3'
	
	R	5'- GAGAGCTCCGTCAAGGGACGTCAGAAAA - 3'

AGRO_0033	F	5'- CGTGACAAGCTTTTCATAGATGCAGGCAACGTC - 3'
	
	R	5'- GAACGTGAGCTCCTGGTGGGCGGATGATTGT - 3'

AGRO_0636	F	5'- CCTAGAAAGCTTCACAATAGTCGCTGCCAAGT - 3'
	
	R	5'- CCTAGAGAGCTCGCTTGTCTCGTGACGCTCATT - 3'

AGRO_1729	F	5'- CGTGACAAGCTTCGGGATGGCAATGGTCTCGT - 3'
	
	R	5'- CTTGTAGAGCTCCTGGCGTTGATACCCATGCT - 3'

AGRO_3967	F	5'- GAGTGTAAGCTTTCCAGCACATAATAGGGCGA - 3'
	
	R	5'- CTTGGAGAGCTCGACACTTGCGGTTGTCATCG - 3'

*Gene knockout confirmation*		

GM^R^	F	5'- GATGCCCATACTTGAGCCACCTAAC - 3'

*nifR*	R	5'- AAGATCATCATTTGCCTCTTCCGGAG - 3'

*ppx1 *(AGRO_2774)	R	5'- GCGAAGCGATCCCGTCGTGGCAAGA - 3'

*ppx2 *(AGRO_2552)	R	5'- GACTCGATCAGAAGCACAGGGGC - 3'

*rpoN*	R	5'- ATGACGCATTTCGAGCTGACGCAGTT - 3'

*rrpP*	R	5'- ATGCGGTGTCCTGTCCGTGGTTTA - 3'

*relA/spoT *(AGRO_1497)	R	5'- CGCTTTCTTTTTGTGGAATATCGCA - 3'

AGRO_0033	R	5'- GTCGTGTTTCAAGTTTCAGGGTCCG - 3'

AGRO_0636	R	5'- GATGTTGGGTCTTTGGAAAAAACCG - 3'

AGRO_1729	R	5'- ATGAATGGTGAAAAGCGGGGGAGC - 3'

AGRO_3967	R	5'- GGTGAATCGCAGATGATGGAATTGAT - 3'

*Quantitative RT-PCR*		

*crdS*	F	5'- ATCCAATTCAGCACAATCTCG -3'
	
	R	5'- ACATATCCCCTTTCCATCAG -3'

*rpoD*	F	5'- GGAAATCCAGAACCTCTCCAC -3'
	
	R	5'- GAACTTGTAACCACGGCGATA -3'

Restriction enzyme sites are underlined. For gene knockout confirmation, the primer homologous to the gentamicin resistance gene (GM^R^) was used along with a primer homologous to the sequence upstream of the target gene

### Fermentation for curdlan synthesis

ATCC 31749 colonies were inoculated into test tubes containing 4 mL of LB media and grown overnight at 30°C with 250 rpm. The inoculum was diluted 1500x in a 500 mL flask of 150 mL LB media. After 17 hours of growth, the cells were concentrated by centrifugation (3000 × g, 10 min, 4°C) to a volume of 30 mL. The concentrated culture was added to 450 mL of minimal fermentation media [30] with 1.4 g/L of KH_2_PO_4 _in an Infors Multifors fermenter system with four parallel 500 mL fermentation vessels. NaOH and HCl (1 M) solutions were used for pH control. Agitation was set at 600 rpm with 1 vvm for the air flowrate, and the pH was controlled at pH 7 for growth. The ammonium level was measured using the Berthelot reaction as described by [31]. After nitrogen exhaustion, 10 mL samples were taken every 24 hours for 5 to 7 days. Curdlan and dry cell weight measurements were performed as described by [30].

### RNA isolation and microarray processing

The RNA was immediately stabilized using Qiagen's RNAprotect Bacteria Reagent and stored at -80°C. RNA was isolated using Qiagen's RNeasy mini kit with on-column DNase digestion (15 mg/mL lysozyme, 15 min for lysis). For samples with high levels of curdlan, the volumes of lysozyme solution, RLT buffer, and ethanol were doubled, and a centrifugation step (5,000 × g for 5 min at 10°C) was added after the addition of RLT buffer. Contaminating genomic DNA was removed using Ambion's TURBO DNA-free kit. RNA quality was determined using Agilent's 2100 bioanalyzer with the RNA 6000 Nano kit. RNA concentration was calculated from OD_260_, measured using the SpectraMax M5 microplate reader with Corning's UV-transparent 96-well microplates. Purified RNA samples were stored at -80°C until analysis.

The subsequent steps described in this paragraph were performed by Gene Logic, Inc. The RNA was converted to cDNA and labeled with either Cy-3 or Cy-5 using the Fairplay III Microarray Labeling kit from Agilent. The microarray is an 8 × 15 k custom DNA microarray from Agilent with probes designed using Agilent's eArray program. The eArray program designed probes for 5,580 of the 5,585 predicted genes in the ATCC 31749 genome with two technical replicates for each gene. For each condition, there are 4 biological replicates with dye swap. A microarray scanner model G2505B from Agilent Technologies was employed for microarray imaging, and Agilent's Feature Extraction 10.5 Image Analysis Software was used to measure the signal intensities.

### Microarray data analysis

The mean local background signal intensity was subtracted from the mean signal intensity for each probe spot on the microarray. The background-subtracted signals were then normalized using a lowess normalization to account for dye-labeling bias. The lowess-normalized data was uploaded into GeneSpring GX 10, and a 75^th ^percentile normalization was applied. The normalized data was then analyzed for statistically significant changes in gene expression using a paired *T*-test with a p-value cutoff of 0.05. Statistically significant genes with more than a 2-fold change in gene expression were manually categorized according to function.

### Curdlan synthesis with knockout mutants

For each mutant, the seed culture was inoculated into two flasks containing 150 mL LB media (100x dilution). The cultures were grown at 30°C and 250 rpm until stationary phase (approximately 12 hours, OD_600 _> 1.5) or the late exponential phase (1.3 < OD_600 _< 1.5) was reached. The cells were collected by centrifugation (3,000 × g and 4°C for 10 min). After one wash step with 10% glycerol, the cell pellets were resuspended in nitrogen-free minimal media, containing 40 g/L sucrose, 0.5 g/L KH_2_PO_4_, 0.5 g/L MgSO_4_•7H_2_O, and 10 mL/L of trace element solution [30] (autoclaved separately), pH 7. Nitrogen-free cell cultures were incubated at 30°C and 250 rpm with sampling at 24 hour intervals. PolyP levels in exponential phase cultures were measured as described previously [32].

### Quantitative RT-PCR

Samples (3 mL) were taken at 24, 72, and 120 hours after the shift to nitrogen-free media. RNA was isolated as described above. DNase treated RNA (2 μg) was converted to cDNA using Invitrogen's Superscript III first-strand synthesis system. Gene expression was analyzed using Applied Biosystems' SYBR green PCR master mix and 7300 Real-Time PCR System. A constitutively expressed sigma factor, *rpoD*, was used for normalization, and primers for *crdS *and *rpoD *measurement are listed in Table 4. The 2-ΔΔC_T _method was applied to analyze the change in gene expression between the wild type and knockout strains, Δ*ppx2 *and Δ*relA/spoT *[33].

## Abbreviations

AA: Amino acid; ADP: Adenosine diphosphate; Alg44: Putative alginate biosynthesis protein; ATP: Adenosine triphosphate; BcsA: Cellulose synthase; Ca^2+^: Calcium ion; c-di-GMP: bis-(3',5')-cyclic-dimeric-guanosine monophosphate; cfu: Colony forming units; DGC: Diguanylate cyclase; EPS: Exopolysaccharide; FleQ: Transcriptional regulator of flagella and PEL synthesis; GDP: Guanosine diphosphate; GlnB: PII protein; GlnD: Nitrogen sensor protein; GlnK: PII protein; GMP: Guanosine monophosphate; GppA: pppGpp phosphohydrolase; GTP: Guanosine triphosphate; H^+^: Proton; NDK: Nucleoside diphosphate kinase; NtrB: Histidine kinase/phosphatase; NtrC: Nitrogen assimilation regulatory protein; PDE: Phosphodiesterase; PelE: PEL exopolysaccharide biosynthesis protein; polyP: Polyphosphate; Pi: Phosphate; PPase: Pyrophosphatase; ppGpp: Guanosine tetraphosphate; PPi: Pyrophosphate; PPK: Polyphosphate kinase; pppGpp: Guanosine pentaphosphate; PPX: Exopolyphosphatase; RelA: (p)ppGpp synthetase; RNAP: RNA polymerase; RpoN: Sigma factor 54; SpoT: ppGpp pyrophosphohydrolase/(p)ppGpp synthetase

## Competing interests

The authors declare that they have no competing interests.

## Authors' contributions

AMR carried out all experiments presented in this work, with the exception of the cDNA library preparation and microarray hybridization which was performed by Gene Logic, Inc. AMR and RRC participated in the design of the study, analysis of the results, and drafting of the manuscript. All authors read and approved the final manuscript.
